# Social penalty promotes cooperation in a cooperative society

**DOI:** 10.1038/srep12797

**Published:** 2015-08-04

**Authors:** Hiromu Ito, Jin Yoshimura

**Affiliations:** 1 Graduate School of Science and Technology, Shizuoka University, Hamamatsu, 432-8561, Japan; 2 Department of Mathematical and Systems Engineering, Shizuoka University, Hamamatsu, 432-8561, Japan; 3 Department of Environmental and Forest Biology, State University of New York College of Environmental Science and Forestry, Syracuse, NY 13210 USA; 4 Marine Biosystems Research Center, Chiba University, Uchiura, Kamogawa, Chiba 299-5502, Japan

## Abstract

Why cooperation is well developed in human society is an unsolved question in biological and human sciences. Vast studies in game theory have revealed that in non-cooperative games selfish behavior generally dominates over cooperation and cooperation can be evolved only under very limited conditions. These studies ask the origin of cooperation; whether cooperation can evolve in a group of selfish individuals. In this paper, instead of asking the origin of cooperation, we consider the enhancement of cooperation in a small already cooperative society. We ask whether cooperative behavior is further promoted in a small cooperative society in which social penalty is devised. We analyze hawk-dove game and prisoner’s dilemma introducing social penalty. We then expand it for non-cooperative games in general. The results indicate that cooperation is universally favored if penalty is further imposed. We discuss the current result in terms of the moral, laws, rules and regulations in a society, e.g., criminology and traffic violation.

Game theory has been formulated in the economic context and applied to biology to solve the question of altruistic behavior in animals and humans^1^,^2^,^3^,^4^. Why animals and humans sometimes behave an altruistic or cooperative behaviors, even if their expected returns (rewards) were minimal compared with their costs of behaviors? For example, a human adult sometimes dive into a raging stream to rescue an unknown (unrelated) child, even if he/she cannot swim. The results are usually the drowned of both the rescuer and the child. The origins of these altruistic and cooperative behavior may be partly explained by kin selection, where the group (society) is formed mostly by kin members^5^,^6^. However, human societies and some highly sophisticated animal societies are formed mostly by unrelated (non-kin) individuals. Cooperative and altruistic behavior in such societies cannot be explained by kin selection and the inclusive fitness theory. Thus the origins of cooperation (and altruism) in an unrelated society (group of non-kin individuals) is a major question in evolutionary game theory^4^. Vast studies in traditional game theory have revealed that in non-cooperative games selfish behavior generally dominates over cooperation and cooperation can be evolved only under very limited conditions, e.g., spatial structures^7^,^8^,^9^. The origin of cooperation is also studied in public goods games. Some studies succeed in explaining the mechanism that cooperation actions evolve from a non-cooperative society by introducing various elements (e.g., spatial interaction and population structure) into public goods game^10^,^11^,^12^,^13^,^14^,^15^.

These studies ask the origin of cooperation: why cooperation could have evolve in a group of selfish individuals. However, the development and diversification of cooperation is a totally different question from the origin of cooperation, when human forms small tribes. In this paper, we specifically ask the further development of cooperation in a small cooperative society. This question explains why a small primitive human cooperative societies could have evolved to become a modern complicated cooperative society. A small cooperative society (tribe) should be devised of moral, law, rules and regulations, some of them with a penalty to keep the cooperative unity of the tribe. We here introduce social penalty for non-cooperative actions in some non-cooperative games to test whether cooperation is further promoted or not. We specifically evaluate the effects of penalty in hawk-dove game and in prisoner’s dilemma game. The results indicate that cooperation is universally favored when penalty is imposed. We thus conclude that the further advancement of cooperation is generally promoted by social penalties in a once-cooperative society, as in most human societies. We discuss the implication of penalty introduction in modern society, with an example of traffic law in Japan^16^,^17^,^18^,^19^,^20^.

## Models and Results

### Hawk-Dove game

**Figure 1:**
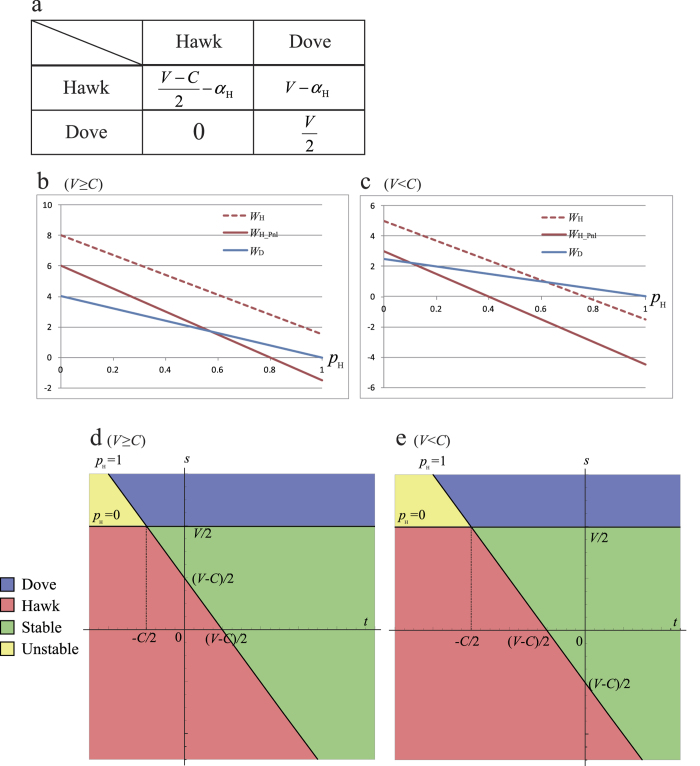
Social penalty introduced into the hawk-dove game. (**a**) Payoff matrix of a hawk-dove game include social penalty *α*_H_. (**b**,**c**) The average payoffs (*W*) of hawk and dove. Values of *W*_H_ (*p*), *W*_H_Pnl_ (*p*), and *W*_D_ (*p*) are plotted against the frequency *p* of hawk. The intersections determine indicates a stable mixed strategy ESS (*t* = 1, s = 2). (**b**,**d**) Fighting between hawk is mild; that is, *V* ≥ *C* (*V* = 8, *C* = 5); (**c,e**) Fighting is severe, *V* < *C* (*V* = 5, *C* = 8). (**d**,**e**) The phase diagram of *t* and *s*. Outcomes depend on penalty parameters: pure Dove (blue), pure Hawk (Red), stable mixed strategy ESS (Green), and non-ESS Nash equilibrium (Yellow).

Hawk-dove game consists of two opposite strategies: (1) hawk H (non-cooperative strategy) and (2) dove D (cooperative strategy). We introduce social penalty (*α*_H_) to hawk strategy in hawk-dove game (Fig. 1). The social penalty reduces the benefit of hawk in the payoff matrix (Fig. 1a). Here we apply social penalty to the modified hawk-dove game. If a hawk opposes to a dove, the hawk gains the pay-off *V*, while the dove receives the pay-off 0 (such as *V* > 0). However, a hawk has to pay a combat cost when it battle with another hawk. Let this cost be *C*. If two hawks oppose each other, the loser pays the combat cost and the winner receives the pay-off *V*. Hence each hawk gains the average pay-off (*V*−*C*)/2. Note that the all hawks receive social penalty (*α*_H_) in this model. Therefore, the pay-off of the hawk becomes the (*V*−*C*)/2-*α*_H_ when they fight against hawk. Similarly, the pay-off of the hawk becomes *V*-*α*_H_ when they fight against dove. Then, when the frequency of hawk is *p* = *p*(H), the fitness of hawk with penalty *W*_H_Pnl_ is given by
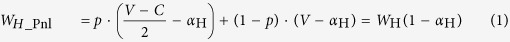
Where the penalty universally reduces *α*_H_ from the payoff of hawk. The fitness of dove *W*_D_ is not different from the traditional hawk-dove game, as
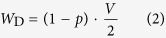


When pure hawk is optimal without penalty (*V* ≥ *C*), the penalty may move the ESS *p*_H_* to the mixed optimum (Fig. 1b). Here hawk in the traditional hawk-dove game (not assuming social penalty) is always the most suitable strategy, because the fitness of a hawk (*W*_H_, dashed line in Fig. 1b) is never be less than that of a dove at every *p*_H_. However, introduced social penalty lowers the fitness of a hawk (*W*_H_Pnl_, solid line in Fig. 1b). With a sufficient level of penalty, the mixed ESS becomes optimal (the intersection in Fig. 1b). When mixed strategy is optimal without penalty (*V* < *C*), the penalty moves *p*_H_* toward more dove (Fig. 1c). If the penalty is large enough, pure dove may become optimal. Note that the mixed ESS *p*_H_* with penalty is given by
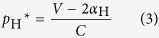
as long as 0 < *p*_H_* < 1. If the calculated *p*_H_* becomes less than zero (<0), pure dove is optimal (*p*_H_* = 0). Similarly if it becomes more than one (>1), pure hawk is optimal (*p*_H_* = 1).

Let social penalty *α*_H_ be a linear function of *p*, such that *α*_H_ = *t* · *p *+ *s*, then the move by penalty can be expressed by a phase plane, where the origin indicates no penalty (Fig. 1d,e). In this phase plane, increasing *t* means moving toward “mixed ESS”; while increasing *s* means moving toward “pure dove”. Thus either coexistence or pure cooperation is promoted depending on the weight on the penalty.

### Prisoner’s dilemma game

**Figure 2:**
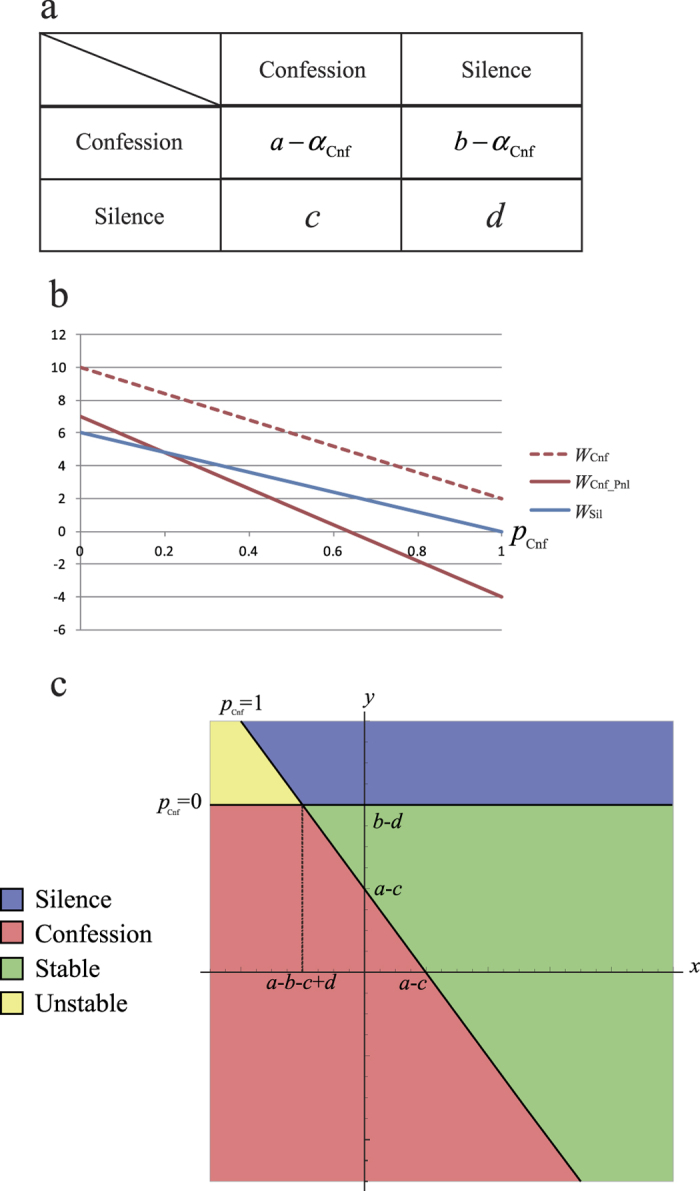
Social penalty introduced into the prisoner’s dilemma game. (**a**) Payoff matrix of a prisoner’s dilemma game include social penalty *α*_Cnf_. (**b**) The average payoffs (*W*) of confession and silence. Values of *W*_Cnf_ (*p*), *W*_Cnf_Pnl_ (*p*), and *W*_Sil_ (*p*) are plotted against the frequency *p* of confession. The intersections determine a stable mixed strategy ESS (*a* = 2, *b* = 10, *c* = 0, *d* = 6, *x* = 3, *y* = 3). (**c**) The phase diagram of *x* and *y*. Outcomes depend on penalty parameters: pure Silence (blue), pure Confession (Red), stable mixed strategy ESS (Green), and non-ESS Nash equilibrium (Yellow).

In prisoner’s dilemma game, we have two strategies: (1) confession Cnf (non-cooperative behavior or deception against the opponent player), and (2) silence Sil (cooperative behavior towards the opponent). We also apply social penalty to the modified prisoner’s dilemma game. We here introduce social penalty (*α*_Cnf_) to confession (Fig. 2). The social penalty reduces the benefit of confession in the payoff matrix, where *b* > *d* > *a* > *c* and *d* > (*b* + *c*)/2 (Fig. 2a). When one suspect who receives an investigation confesses, he/she receives the prison term pay-off with social penalty (*a*−*α*_Cnf_) when another accomplice also confessed. Similarly, a suspect who selected confession receives the prison term pay-off with social penalty (*b*−*α*_Cnf_) when another accomplice keeps silent. If he/she keeps silent, we suppose that he/she is not exposed to social penalties. Then, when the frequency of confession is *p* = *p*(Cnf), the fitness of confession with penalty *W*_Cnf_Pnl_ is given by
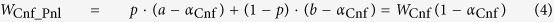
where the penalty universally reduces *α*_Cnf_ from the payoff of confession. The fitness of silence *W*_Sil_ is not different from the traditional prisoner’s dilemma game, as
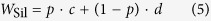


When pure confession is optimal without penalty, the penalty may move the ESS *p*_Cnf_* to the mixed optimum, if the penalty *α*_Cnf_ is large enough (Fig. 2b). Here confession in the traditional prisoner’s dilemma game (not assuming social penalty) is always the most suitable strategy, because the fitness of a confession (*W*_Cnf_, dashed line in Fig. 2b) is never be less than that of silence at every *p*_Cnf_. However, introduced social penalty lowers the fitness of a confession (*W*_Cnf_Pnl_, solid line in Fig. 2b). With a sufficient level of penalty, the mixed ESS becomes optimal (the intersection in Fig. 2b). Furthermore, pure silence strategy Sil may become optimal, if *α*_Cnf_ is much larger. Here the ESS *p*_Cnf_* with penalty is given by
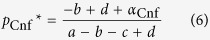
as long as 0 < *p*_Cnf_* < 1. If the calculated *p*_Cnf_* becomes less than zero (<0), pure Sil is optimal (*p*_Cnf_* = 0). Similarly if it becomes more than one (>1), pure Cnf is optimal (*p*_Cnf_* = 1).

Let social penalty *α*_Cnf_ be a linear function of *p*, such that *α*_NC_ = *x* · *p *+ *y*, then the move by penalty can be expressed by a phase plane, where the origin indicates no penalty (Fig. 2c). In this phase plane, increasing *x* means moving toward “mixed ESS”; while increasing *y* means moving via “mixed ESS” to “pure silence”. Thus either coexistence or pure cooperation is promoted depending on the weight on the penalty.

### Non-cooperative games in general

The current results can be generalized easily for most non-cooperative games involving cooperators C and non-cooperators NC. We could say that penalty on non-cooperative action always promote cooperation under social punishment settings. In the previous two example, penalty is imposed on any action of non-cooperators, irrespective of the opponent. If penalty is imposed only on an action against one opponent (e.g., either Hawk or Dove opponent, but not both), the above results still hold. We here impose a penalty to non-cooperators when they play against one or more types of opponents. Let *p*_cal_* be the calculated ESS without penalty, such that
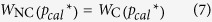
where *W*_NC_(*p*_cal_*) and *W*_C_(*p*_cal_*) are the fitness of NC and C, respectively. Then we have two cases depending on the value of *p*_cal_*: (1) 0 ≤ *p*_cal_* ≤ 1; and (2) *p*_cal_* ≥ 1.

(1) In case of 0 < *p*_cal_* ≤ 1.

**Figure 3:**
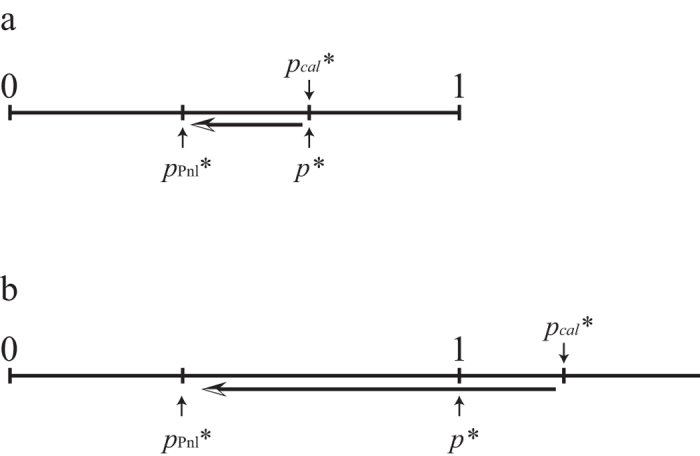
Comparison between conventional ESS and ESS with penalty. (**a**) The case of calculated ESS without penalty lays between 0 and 1 (*p*_cal_* = p*). (**b**) The case of calculated ESS without penalty exceeds 1 (*p*_cal_* > p* = 1).

In this case, p* = *p*_cal_*. Therefore, always *p*_Pnl_* < p*, because *W*_NC_(*p*_Pnl_*) < *W*_NC_(p*), where *p*_Pnl_* and p* are the ESS with/without penalty, respectively. This means that the mixed equilibrium always move toward more cooperators (Fig. 3a). If penalty is sufficiently large, pure cooperators become the ESS.

(2) In case of *p*_cal_* ≥ 1.

In this case, p* = 1. Therefore, if penalty on non-cooperator *α*_NC_ is sufficiently large, we get *p*_Pnl_* < p* (Fig. 3b); otherwise *p*_Pnl_* = p*.

## Discussion

The current results demonstrate that the introduction of penalty is likely to promote cooperation and never reduce cooperation. This results can be applicable to the criminological aspect of laws and regulations in the current legal systems of a modern society. A sound example is the recent changes in the Road Traffic Law of Japan following the fatal accidents caused by drunken driving^19^,^20^. In this case, two malicious accidents lead to the toughening the traffic law. First, in the accident on a highway in Tokyo in 1999, two young children ( aged 1 year and 3 years) killed by a heavily drunken truck driver. Second, in the accident on a bridge at Fukuoka in 2006, a car driven by a heavily drunken driver pushed a car with a whole family off the bridge into water, and three young children (aged 1, 2, and 3 years) were drowned. These two crashes were reported sensationally as a serious social problem and arouse out of discussion among Japanese citizen that the penalty of drunken driving at that time was too light. The signature collection campaign was performed by the bereaved of the victims who lost their life by these habitual and/or vicious drink-driving, leading to the establishment of new laws. The Road Traffic Law of Japan was revised in 2001 and 2007 and applied in 2002 and 2007, respectively. After these revisions in traffic penalty, the rate of accidents caused by drunken driving decreased radically in Japan^16^,^17^,^18^,^19^,^20^.

It is important to point out that the current model assumes the society governing the penalty is independent from the players. In public goods game^10^,^11^,^12^,^13^,^14^,^15^,^21^,^22^,^23^,^24^,^25^, Axelrod introduced norm game^10^ to evaluate whether punishment affected promotion of cooperation behavior. Later, spatial interaction^26^ were introduced in evolutionary game theory^7^,^8^. Recently spatial interaction was shown to promote cooperation in public goods game^11^,^12^,^13^,^14^. Similarly, population structures were shown to promote cooperation^12^,^14^,^15^. These studies suggest that cooperation is promoted with the negative feedback of punishment in non-cooperative public goods games, if spatial interactions and/or population structures are built in the model. This also implies that cooperation is hard to evolve if no spatial or population structures are added in a public goods game. In our model of cooperative games (assuming the establishment of cooperative society), cooperation is easily promoted without these additional structures. It is interesting whether cooperation is promoted in a public goods game in the current cooperative society.

Our model is applicable to the laws, regulations in the modern society. For example, the game conditions of the above traffic example can be summarized as follows:

1. All cooperators except the bereaved families does not pay the individual costs for public goods compared with non-cooperators, but agree to toughen the law.
2. Both cooperators and non-cooperators pays the cost of public goods evenly as a form of taxation to maintain the laws of the society.
3. All players have no power to decide whether he/she choose to pay or not the cost of public goods.
4. All players cannot decide the decision of penalty revisions.

As in this examples, the laws and regulations are indirectly determined in the society. These points contradicts with the basic assumptions used in the public goods game. In our models, we assume the laws and regulations given by the society independent of the individual players. As an extension of the current model, the society may be added as a feedback unit that is indirectly governed by all the players, e.g., voting system^27^,^28^. The role of media may be important here in the bridge between the public and the government^19^,^29^,^30^.

**Figure 4:**
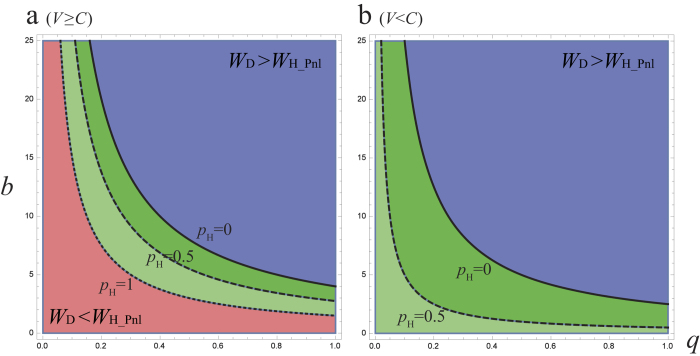
Phase plane of arrest ratio *q* and sentence *b* in hawk-dove game with social penalty. (**a**) Fighting between hawk is mild; that is, *V* ≥ *C* (*V* = 8, *C* = 5). (**b**) Fighting is severe, *V* < *C* (*V* = 5, *C* = 8). Three different lines indicate the optimal sentence b* (Equation 8) for the equilibrium condition (*W*_D_ = *W*_H_): *p*_H_ = 0 (solid), 0.5 (dashed), and 1 (dotted). Equilibrium is shown as follows: pure Dove (blue), pure Hawk (Red), mixed ESS (light and dark Greens).

In our results, the density of non-cooperator is affected by the penalty of criminal action. Here the crime penalty *α*_Sus_ of a suspect depends not only on plausible sentences, but also on arrest ratios (the probability of arrests), such that 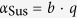, where *b*(≥0) and *q*(0 ≤ *q* ≤ 1) are sentence and arrest ratio. If we apply this relationship to hawk-dove game with *V* ≥ *C* (Fig. 1), the equilibrium sentence b* becomes (Fig. 4):
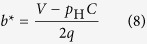


This means that, if the arrest ratio is very low, the criminal activities cannot be reduced even by severe sentences, such as death penalty and lifetime sentences. However, if the arrest ratio is sufficiently high, light sentences is enough to reduce them^17^,^18^,^19^,^20^,^31^,^32^.

In the current model, we only consider punishment against cooperation. In spatial public goods games, not only punishment, but also reward is often considered. In these studies, the correlation between punishment and reward reciprocity (positive and negative feedbacks) were shown to be ineffective compared with punishment or reward alone^13^. It is interesting whether these results holds in the current cooperative game settings.

In our model, we do not question the origin of cooperation. We specifically ask whether cooperation is further promoted in a modern large cooperative society consisting of non-kin people. In contrast, cooperation may be originated in a small society consisting of mostly, if not all, kin members. We should stress that the origin and the successive development of societies are two different problems.

## Additional Information

**How to cite this article**: Ito, H. and Yoshimura, J. Social penalty promotes cooperation in a cooperative society. *Sci. Rep.* **5**, 12797; doi: 10.1038/srep12797 (2015).
